# The Doping of a Carbon Coating with Phosphorus as a Potential Way of Improving the Biological Properties of Diamond-like Carbon

**DOI:** 10.3390/ma17235859

**Published:** 2024-11-29

**Authors:** Krzysztof Jastrzębski, Jacek Grabarczyk, Piotr Niedzielski, Anna Jędrzejczak, Anna Sobczyk-Guzenda, Witold Szymański, Marta Kamińska, Beata Skibska

**Affiliations:** 1 Institute of Materials Science and Engineering, Lodz University of Technology, Stefanowskiego 1/15, 90-537 Lodz, Poland; jacek.grabarczyk@p.lodz.pl (J.G.); piotr.niedzielski@p.lodz.pl (P.N.); anna.jedrzejczak@p.lodz.pl (A.J.); anna.sobczyk-guzenda@p.lodz.pl (A.S.-G.); witold.szymanski@p.lodz.pl (W.S.); marta.kaminska@p.lodz.pl (M.K.); 2 Department of Applied Pharmacy, Faculty of Pharmacy, Medical University of Lodz, Jana Muszyńskiego 1, 90-001 Lodz, Poland

**Keywords:** P-DLC, biomaterial, RF-PACVD, osteogenesis, hemocompatibility, bactericidal effect

## Abstract

The potential of diamond-like carbon coatings in medicine can be increased by doping them with various elements. Such modifications especially affect the biological properties of the synthetized films. In the following research, phosphorus was introduced into the carbon matrix by means of the chemical vapor deposition technique and using an organic precursor. With the addition of about 1.6 and 4.3 at% of dopant, not only was the surface roughness increased, but significant changes in both the mechanical and biological properties were also observed. The presence of phosphorus reduced the hardness of DLC coatings but still improved this parameter in comparison to the substrate material—AISI316LVM. A biological examination revealed the bacteriostatic potential of doped coatings regardless of their chemical composition. Increasing the amount of phosphorus improved the proliferation of osteoblasts (*Saos-2* cell), but the opposite effect was achieved for the endothelial cell line (*EA.hy926).* Another important aspect is the reduction in platelet activation, especially for low amounts of dopant.

## 1. Introduction

Diamond-like carbon (DLC) is an interesting material for application as a thin layer on biomaterials such as AISI316LVM steel and alloys of titanium or cobalt [1,2]. The main task of DLC coatings is to improve the physicochemical and biological properties of materials, including, among others, by increasing corrosion resistance, changing the tribological properties, and limiting the release of metal ions in order to protect/limit the occurrence of allergies. The greatest potential in the use of DLC coatings is visible in various fields of medicine, such as orthopedics (intramedullary nails, hip joint prostheses), neurosurgery (spinal fusions), maxillofacial surgery (mandibular fusions), or cardiac surgery (heart valves, stents) [1,3,4]. The number of procedures with the potential for surface modifications related to orthopedics alone is estimated in Europe to be approximately 1.5 million [5]. Specific mechanical properties, such as increased abrasion resistance, high hardness, a low coefficient of friction, and high chemical stability, have also resulted in the use of DLC layers as protective films for surgical instruments.

The evolution of the concept of DLC is naturally related to the doping of films with various elements. The first attempts to introduce admixtures to such carbon matrices were related to their application in electronics, where boron atoms caused a change in electrical properties in p-type semiconductors and nitrogen and phosphorus in n-type semiconductors. Over time, such modifications (especially those related to altering surface properties) also seemed promising in medical applications, mainly in terms of improving/inducing bactericidal, anti-inflammatory, and biocompatibility properties in materials. DLC coatings doped with silver (Ag-DLC) are known to limit the development of harmful microorganisms, and their inhibition of bacterial multiplication occurs after just a few hours [6,7]. A similar effect is also noticeable when copper is used as an admixture [8]. Each element present in the carbon matrix can tailor the properties of the final coating towards specific applications, yet it is important to determine not only the dopant itself but also its concentration.

Phosphorus performs many valuable functions in the body. Its presence, constituting approximately 1% of human weight, contributes to the proper functioning of the skeletal system (approximately 85% of the content of this element in the body is related to the skeletal system), heart muscle cells, and the neuromuscular system [9]. 

The osteogenic properties of compounds containing phosphorus are related, among others, to bone mineralization (together with calcium, it increases the proliferation and viability of osteoblasts on modified implant surfaces), cell signaling (inorganic phosphate changes the early stages of osteogenic differentiation by influencing the Wnt signaling pathway [10]), or influencing the acceleration of osteoblast adhesion (through the adsorption of large amounts of fibronectin and vitronectin) [11,12,13]. Phosphorus-doped carbon layers (P-DLC) have already been investigated in medical applications [14] as implantable devices in the human nervous system. The properties of phosphorus itself, as well as its application in carbon coatings, puts this element alongside dopants such as Ag, Ca, Si, Cu, and many more, with the potential for doping the surface of metallic implants.

The following article focuses on a general investigation of the properties of P-DLC coatings for applications in biomedicine, for example, as implants in the skeletal or circulatory system. The impact of this investigation will be the initial verification of the biological potential of such coatings. This investigation covers, among other things, the assessment of P-DLC’s osteogenic bactericidal and thrombogenic character. 

## 2. Materials and Methods

**Sample preparation and coating synthesis**

The RF-PACVD (radio-frequency plasma-activated chemical vapor deposition) method was used to produce P-DLC samples on AISI316 LVM substrate materials. Samples in the form of cylinders 6 mm high and 16 mm in diameter were mechanically grinded, mirror-polished, and degreased by ultrasound washing for 10 min in organic solvent (acetone). During deposition, methane was used as the carbonaceous gas, while doping was achieved with the liquid phosphorus precursor trimethyl phosphate ((CH_3_)_3_PO_4_) (Sigma Aldrich, Saint Louis, MO, USA). The deposition process was initiated by etching for 10 min at a temperature of about 450 °C with a negative polarization potential of 1100 V and a pressure of 20 Pa—the methane flow was equal to 10 sccm. The synthesis step was carried out at a pressure of 30 Pa (10 sccm of methane flow) for 2 min at a temperature of 470 °C and under a negative polarization potential of 900 V and then for 1.5 min at a temperature of 400 °C and a negative polarization potential of 400 V. The vapors of the precursor of phosphorus, heated to 37 °C, were introduced into the working chamber via a mass flowmeter. The flow was 20 sccm or 30 sccm for the samples denoted as P-DLC1 and P-DLC2. As a reference, coatings produced under the same conditions as their doped counterparts, but with only a methane flow, were also prepared.

**Sample sterilization**

Prior to further examination, all the samples were steam-sterilized. Autoclaving was performed within 15 min at 121 °C. The equipment used was Classic Prestige Medical (Blackburn, UK).

**Chemical composition**

Characterization of the chemical composition of the synthesized coating was performed in a complex manner involving the following: the direct examination of elemental content using X-ray Photoelectron Spectroscopy (XPS) and the verification of bond systems using Fourier transform infrared (FTIR) and Raman spectroscopy. Finally, the effect of the coating on the hydrophilicity/hydrophobicity of the surface was verified by evaluating surface free energy using the sitting drop method.

**XPS**

The examination of surface chemistry using XPS involved quantitative chemical analysis, based on wide spectra up to 1200 eV (the data acquisition time, dwelling time, and energy step were equal to, respectively, 240 s, 1000 ms, and 1000.0 meV). In the case of narrow spectra, the dwelling time was equal to 250 ms and the energy step was 100 meV). Additionally, the peak of carbon (C1s) was analyzed by means of deconvolution using Gauss–Lorentz curves in PeakFit 4.12 software, (SeaSolve Software Inc., San Jose, CA, USA). Characteristic peaks with maxima corresponding to the following bond energies were taken into account: 284.5 eV, 285.3 eV, 286.4 eV, 287.5 eV, and 288.7 eV. These were related to C=C; C–C; C–O; C=O; and COOR bonds. The above-mentioned assessment was performed using the AXIS Ultra DLD (Kratos Analytical, Manchester, UK) system and 3 randomly selected spots on the surface of each sample.

**FTIR**

A Nicolet iS50 spectrometer (Thermo Scientific, Waltham, MA, USA) equipped with a DRIFT adapter (Harrick Scientific, Pleasantville, NY, USA) and operating in absorbance mode was used to perform FTIR analysis at room temperature. The main working parameters were a spectral range equal to 4000 to 500 cm^−1^, a resolution of 4 cm^−1^, 120 measurement cycles, and an angle of 60°. The level of noise was equal to 0.1 (Voigt mode). Spectrum deconvolution was performed in OMNIC 9.16 software (Thermo Scientific, Waltham, MA, USA).

**Raman spectroscopy**

Raman spectra were recorded at room temperature using an InVia (Renishaw, Gloucestershire, UK) Raman microscope with a backscatter geometry (532 nm laser wavelength) and laser power of 0.3 mW. The measurements were repeated three times for each analyzed material in randomly selected areas of the coatings. All the obtained spectra were deconvoluted using the Gaussian function using PeakFiT 4.12 software (SeaSolve Software Inc. San Jose, CA, USA). The obtained results were averaged.

**Surface free energy (SFE)**

The polar and dispersive components of surface free energy were evaluated based on the Owens–Wendt method and Easy Drop system (Krüss GmbH, Hamburg, Germany) using Drop Shape Analysis 1.90.0.14 software (Krüss GmbH, Hamburg, Germany). This requires measurements of the contact angles of sitting drops of distilled water and diiodomethane with an accuracy equal to 0.1°. The analysis was performed each time in triplicate for both testing liquids.

**Surface morphology and topography**

Roughness evaluation was performed using a multi-mode atomic force microscope (AFM) equipped with a Nanoscope V controller (Bruker Corporation, Billerica, MA, USA). The measurements were conducted at room temperature in triplicate. In each case, the Intermittent contact mode was used to prepare 512 scan lines that corresponded to a 10 μm × 10 μm area or a 1 μm × 1 μm area. NanoScope 7.0 (Veeco, Plainview, NY, USA) and MountainMap 5.0 (Digital Surf, Besancon, France) software were used for data acquisition and image processing.

**Mechanical properties**

The evaluation of mechanical properties comprised hardness, adhesion, and wear tests conducted on a nano-scale at room temperature in triplicate. Both measurements were conducted using a Nano Indenter G200 (MTS NANO Instruments, Oak Ridge, TN, USA). As the penetrators, we used a Berkowits penetrator (for measuring nanohardness) and a diamond cone (for other measurements) with an apex angle equal to 60° and rounding of 1 μm. Microhardness was performed in continuous stiffness mode. The evaluation of adhesion followed the scratch test protocol where the adhesion force was assessed on the basis of microscopic observations, stepwise variation in the coefficient of friction, and changes in the penetration depth of the indenter in the tested material. The wear tests were based on linear reciprocating movements conducted at a frequency of 1 Hz, and the total wear path was 20 m. The volumetric wear was calculated on the basis of the wear index, and surface abrasion was determined according to the transverse profiles of the obtained groves. All the measurements were performed using MountainMap 5.0 (Digital Surf, Besancon, France).

**Biological response**
**Microbial colonization**

LIVE/DEAD staining of the model organism *E.coli* was used to assess the bactericidal potential of the manufactured coatings. Measurements were conducted for liquid cultures of bacteria (Luria Bertani Broth) incubated with the tested samples for 24 h at 37 °C. The double staining protocol involves the usage of bis benzamidine (Molecular probes, excitation at wavelength of 345 nm) and propidium iodide (molecular probes, excitation at wavelength of 538 nm). After 10 min of incubation with both dies in the dark, the surface of the samples was investigated using fluorescence microscopy (Olympus GX71, Tokyo, Japan). For each sample, 5 photos were taken at various random locations. Stained cells (green—live and red—dead) were counted based on open-source Image J 1.54k software. As a control, the uncounted AISI316 LVM samples were used.

**Mammalian cell viability**

Two cell lines were investigated: *Saos-2* and *EA.hy926* cultured in consecutive McCoy’s medium (with 15% of fetal bovine serum) and Dulbecco’s modified Eagle’s medium (with 10% of fetal bovine serum). The *EA.hy926* (ATCC^®^ CRL2922™) and *Saos-2* (ATCC^®^ HTB85™) were provided by LGC Standards Ltd. (Middlesex, UK). In the case of both mammalian cell lines, the LIVE/DEAD protocol was applied, involving staining with Hoechst 33342 (Molecular Probes, Eugene, OR, USA—excitation at wavelength of 345 nm), calcein AM (Santa Cruz Biotechnology, Dallas, TX, USA - excitation at 495 nm), and propidium iodide (Molecular Probes, Eugene, OR, USA—excitation at wavelength of 538 nm). The tested materials in 12-well plates were seeded with about 12 × 10^4^ cells of the given cell line per well in 2 mL of the required medium. After 48 h of incubation in standard conditions, the medium was removed, and the cells were washed with a phosphate buffer. After 15 min of incubation of the samples in the dark with a mixture of dies, the cells on the samples were visualized using an InCell Analyzer 2000 automated microscope (GE Healthcare, Chicago, IL, USA). For each sample, 5 pictures in random spots were taken. The calculations of cells (live—green, dead—red, and total—blue) were conducted using InCell Investigator v1.5 (GE Healthcare, Chicago, IL, USA) software.

**Response to contact with blood (thrombogenicity assessment)**

An in vitro thrombogenicity assessment was performed on blood from healthy volunteers who had not taken antiplatelet drugs in the two weeks before donation. The test samples, just after steam sterilization, were exposed to fresh blood for 1 h, providing mild blood flow at 37 °C. Sodium citrate was used as an anticoagulant, and blood was contacted with the sample 10 min after collection from the donor. The samples were then washed three times in phosphate buffer (ATCC) at pH 7.4 and fixed in 2.5% glutaraldehyde (SERVA Electrophoresis GmbH, Heidelberg, Germany ) at 4 °C for 1 h. The samples were again washed with a PBS buffer and dehydrated in ethanol at increasing concentrations (50−100%). Excess alcohol was evaporated at room temperature. The surfaces of the samples thus prepared were sputtered with a 5-nanometre gold layer in a LEICA AM ACE600 sputtering machine (Leica Microsystems, Wetzlar, Germany)and imaged using a JEOL JSM-6610LV (Tokyo, Japan) scanning electron microscope (SEM). A minimum of 10 images were taken at randomly selected locations. Platelets were counted using ImageJ software.

## 3. Results and Discussion

### 3.1. Chemical Composition

The chemical composition of the samples examined through XPS shows, in addition to the dopant elements, carbon and oxygen (see Table 1). The amount of admixture in P-DLC1 and P-DLC2 was as follows: 1.64 ± 0.10 and 4.26 ± 0.23 at%. Such amounts are in line with, for example, the results presented by Dey et al. [15], who used a combination of the RF plasma technique and phosphorus evaporation to obtain a coating containing 2 to 7 at.% of this admixture. 

**Figure 1:**
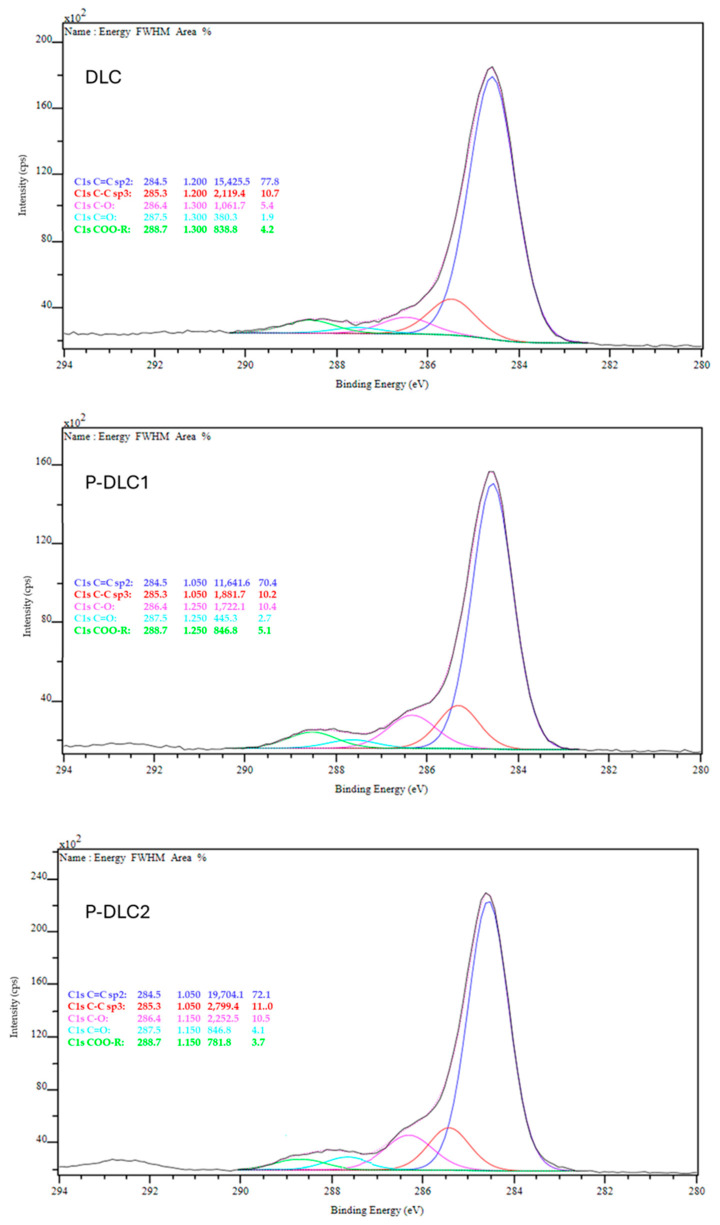
Exemplary spectra of C1s and their deconvolution according to the positions 284.5 eV, 285.3 eV, 286.5 eV, and 288.7 eV, related, respectively, to C=C; C−C; C−O; C=O; and COOX bonds.

In all the analyzed samples, there is a significant number of bonds between carbon and oxygen, which proves the high reactivity of the coating after the deposition process. A similar trend (of rising oxygen content or number of bonds between coating constituents and oxygen) is visible for other types of doped DLC coatings manufactured by similar techniques, like Si-DLC [16], and Cr-DLC [17], as well as Ti-DLC, Al-DLC, and V-DLC [18]. Free bonds on the surface are quickly saturated with oxygen from the atmosphere during chamber aeration. The use of phosphorus as a dopant was associated with an increased number of carbon–oxygen bonds (see Table 2, Figure 1). There is also a clear decrease in the number of sp2-hybridized bonds for P-DLC coatings. The addition of more phosphorus results in a slight increase in this kind of bond. This effect is opposite to that presented by Dey et al. [15]. The number of sp3-hybridized bonds shows lower variations, and only a slight increase can be observed in the case of the P-DLC1 sample.

**Figure 2:**
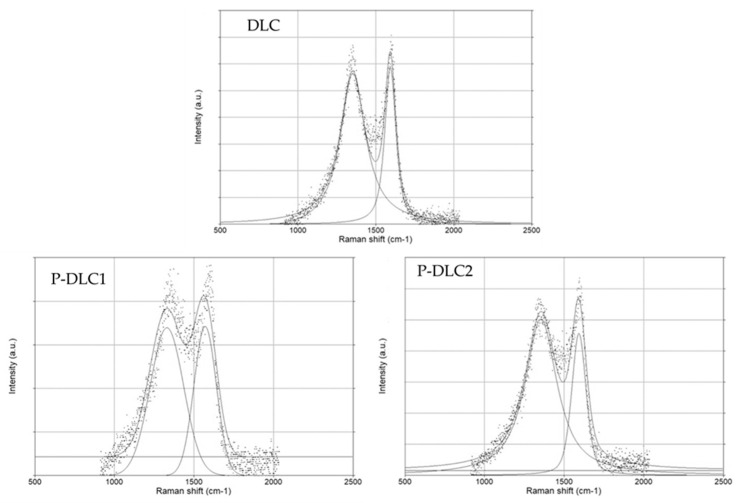
Exemplary Raman spectra of the examined samples with their deconvolution.

The results of the deconvolution of the Raman spectra are presented in Figure 2. The increasing Id/Ig ratio visible due to Raman spectroscopy is related to the rearrangement of sp2 carbon clusters and the transition from an sp3 bond to an sp2 bond. The opposite was true for the coatings of Gao et al. [19], but in that case, the deposition method was different. The increase in the FWHMG (see Table 3) value informs us about the increase in disorder in the chemical structure—i.e., as a result of the changes in the content of carbon bonds with sp3 and sp2 hybridization and changes in the size of clusters. Other research teams have also claimed that the increasing disorder in DLC coatings is correlated with dopant levels [18]. The observed shift in the G peak towards lower wavenumbers may indicate a decrease in the size of sp2-hybridized carbon bond clusters. It may also point to changes in the stress level in the coating matrix. As shown by Batory et al. [20] in the publication on Ag-DLC, the shift in the G band was affected by the size of the admixture agglomerates, which could have resulted in high stress values.

**Figure 3:**
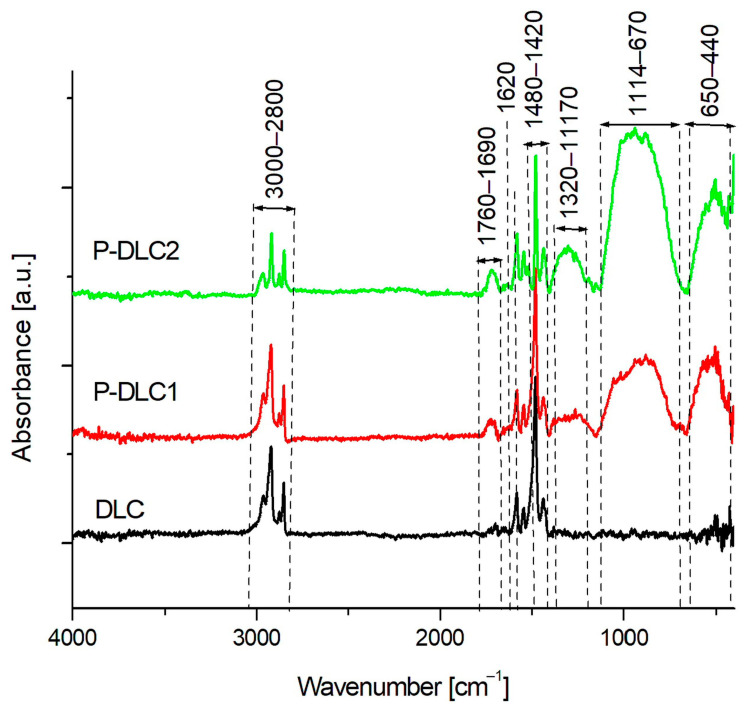
FTIR spectra of DLC, P-DLC1, and P-DLC2 coatings in the range of 4000–e400 cm^−1^.

Figure 3 shows the spectra of the DLC coating and the coatings with phosphorus additive (P-DLC1 and P-DLC2). In all the obtained spectra, clear peaks originating from methyl and methylene groups are visible. However, in the P-DLC2 coating with the highest phosphorus content, the number of these groups decreases slightly. The bands belonging to these groups are visible in two ranges of 3000–2800 cm^−1^ and 1480–1440 cm^−1^. The most interesting region to investigate in the spectra for these groups is in the range of 3000–2800 cm^−1^. In the first range, there are peaks at 2964, 2926, 2876, and 2850 cm^−1^, which belong to the stretching vibrations asym. CH_3_; as. CH_2_; sym. CH_3_; and sym. CH_2_, respectively. In the second range, peaks originate from the deformation vibrations of the CH_2_ and CH_3_ groups. In all the coatings, C=O bonds are present, as evidenced by the presence of a band in the wavenumber range of 1760–1690 cm^−1^. The number of these bonds is greater for coatings with phosphorus added and increases with its increasing concentration in the coatings. At 1620 cm^−1^, there are stretching vibrations of C=C bonds [21]. The intensities of these bonds are very similar regardless of the type of coating. At 1580 cm^−1^, there is a peak most likely belonging to stretching C–O vibrations in the –COOR groups. The next bands that have been identified are found only in coatings with phosphorus added. The first broad band is in the wave number range of 1320–1170 cm^−1^. In this range, there are stretching vibrations originating from P-CH_3_ groups. For them, the most characteristic range is 1320–1280 cm^−1^. In turn, the second part of this broad band, located in the range of 1280–1170 cm^−1^, contains peaks originating from the P=O group [22,23]. In this case, the number of P-CH3/P=O groups increases in the P-DLC2 shell compared to the P-DLC1 shell. Another broad band is located in the range of 1114–670 cm^−1^. In this range, there are stretching vibrations of the P-O and P-O-P groups, but also, at wave numbers of about 1050 and 740, there may be peaks originating from sym. and asym. vibrations of P-O-C bonds. In turn, the band 650–440 cm^−1^ belongs to P=O stretching and P-O deformation vibrations [22,23,24]. The intensity of all three bands increases with the content of phosphorus in the films.

### 3.2. Mechanical and Surface Properties

**Figure 4:**
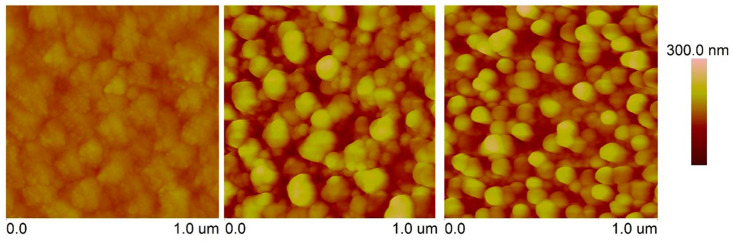
Graphical visualization of surface roughness of examined coatings measured using AFM (from left to right: DLC, P-DLC1, P-DLC2).

Doping carbon coatings with phosphorus is associated with an increase in their roughness (see Table 4). Such results are in line with those presented by S. Wan et al. [25]. As shown in the work of Jui-Yang Lai et al., surface roughness at the cellular level may have a significant impact on the therapeutic properties of coatings [26], including osseointegration. This is visible in the values of the Ra parameter obtained as a result of scans carried out for both the 10 × 10 um and 1 × 1 um areas (Figure 4).

**Figure 5:**
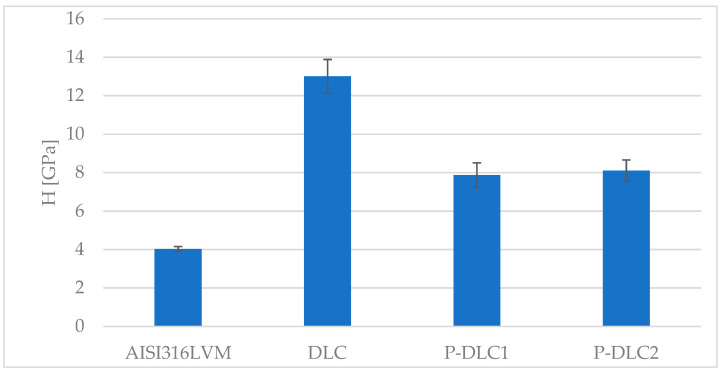
Effect of surface modification on microhardness of examined coating.

The modification of stainless steel with any type of examined coating leads to an increase in surface hardness (see Figure 5) by at least 30%. In the examined range of dopant concentration, the amount of admixture does not differentiate the hardness of P-DLC1 and P-DLC2 coatings. This may be related to there being almost no change in the sp3/sp2 ratio denoted by the XPS examination. Nevertheless, a drop in hardness is observed in comparison to pure DLC. Publications concerning P-DLC coatings usually focus on their electrical or biological features and not their mechanical properties such as hardness. Gao et al. [19] obtained about a 30% improvement in hardness after doping DLC with phosphorus, which is in opposition to the presented results.

What is more, for example, Lu et al. [27] compare the mechanical results of only doped coatings (with oxygen and phosphorus) and not DLC. The doping of carbon obviously affects the hardness of coatings, but the achieved results vary for different admixtures. The deterioration of hardness is visible, for example, for the incorporation of silicon and fluorine into the carbon matrix [28], as well as silver [29], while among the dopants improving this parameter are Ti, Al, and V [18].

**Figure 6:**
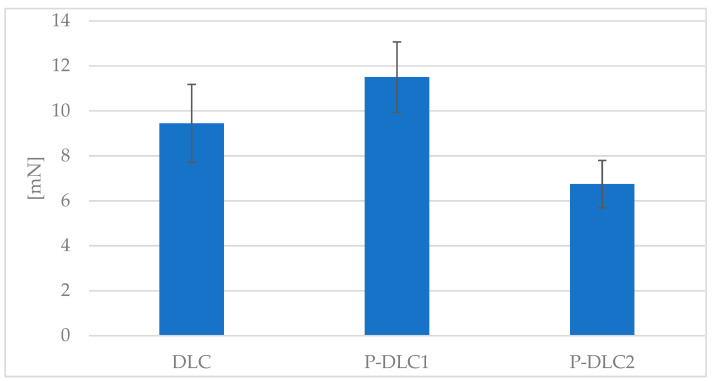
The effect of surface modification on the force of adhesion of the examined coating, evaluated by scratch test.

An analysis of the critical force of delamination at the nano-scale via a scratch test can be used to assess the adhesion of doped carbon coatings, as presented in Figure 6. Small amounts of admixture allowed us to raise this parameter from 9.5 mN to 11.5 mN. The coatings with the highest examined phosphorus content had the lowest adhesion.

**Figure 7:**
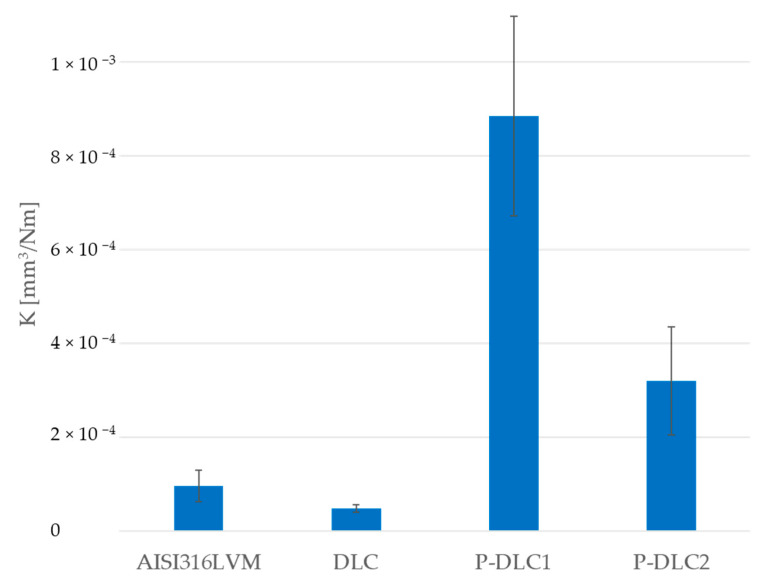
Changes in wear rate of examined samples.

The presence of the DLC coating has a positive effect on the reduction in the wear factor by about 50% in comparison to the substrate material (see Figure 7). Unfortunately, doping significantly worsens this parameter—by about 8 times for P-DLC1 coatings and 3 times for P-DLC2 coatings.

### 3.3. Surface Energy and Wettability

**Figure 8:**
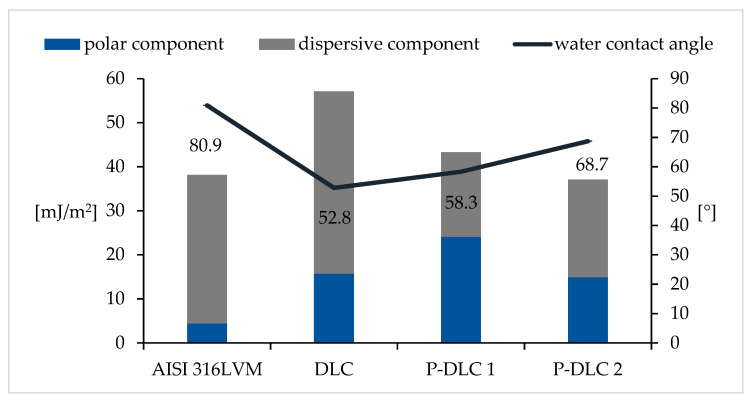
The surface energy and water contact angle of the coatings (only the values of water contact angle are depicted in the figure).

The presence of a coating led to an increase in the total surface free energy, only in the case of P-DLC2, it was on the level of the substrate material. Changes in the chemical structure of DLC, P-DLC1, and P-DLC2 coatings translate directly into the results obtained from the surface wettability tests. The sample of undoped DLC had the highest surface energy (see Figure 8). In the case of the DLC coating, a large share of its structure is made up of non-polar groups such as -CH_3_ and -CH_2_-, which resulted in a high value of the dispersion component SFE, which, in this case, was 41.4 mJ/m^2^, and it is the value of this component that led to an increase in the total value of SFE, which, in this case, reached a fairly high value of 57.1 mJ/m^2^. The addition of phosphorus in the P-DLC1 coating caused an increase in the number of C=O groups, but also influenced the appearance of new bonds in the chemical structure of the P=O, P-O type, which led to a significant decrease in the dispersion component in favor of the polar component, which, in this case, was 24.1 mJ/m^2^, which, in turn, resulted in a decrease in SFE to a value of 43.3 mJ/m^2^. A further increase in the amount of phosphorus in the P-DLC2 coating caused the appearance of a greater number of P-CH3 bonds compared to P=O bonds, which most likely caused a decrease in the polar component to a value of 14.9 mJ/m^2^ and a slight increase in the dispersion component to a value of 22.2 mJ/m^2^. The total SFE value was still close to the value obtained for the P-DLC1 coating.

The presence of phosphorus in the carbon matrix resulted in a decrease in the value of the dispersion component. The change in the values of free energy components occurring as a result of doping is in accordance with the publication of Kwok et al. [30]. The increase in the polar component of sterilized samples is related to both the presence of the admixture itself and the oxidation of the surface.

The use of a carbon coating on the AISI316 LVM substrate reduced the value of the contact angle. With the increasing concentration of phosphorus, the contact angle increased. The obtained values were significantly higher than those presented for P-DLC coatings deposited on silicon [30].

### 3.4. Biological Effect

**Figure 9:**
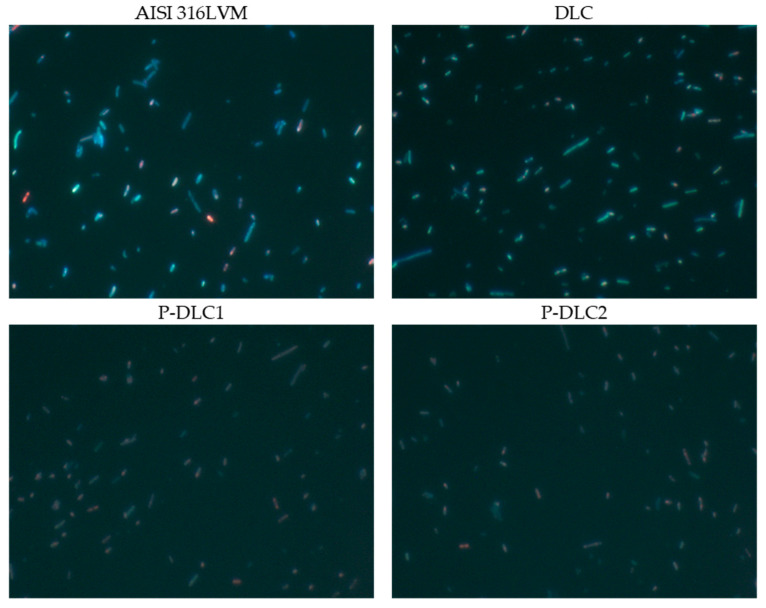
Exemplary microscopic images used for the sake of the evaluation of the bactericidal/bacteriostatic effect of the examined materials.

**Figure 10:**
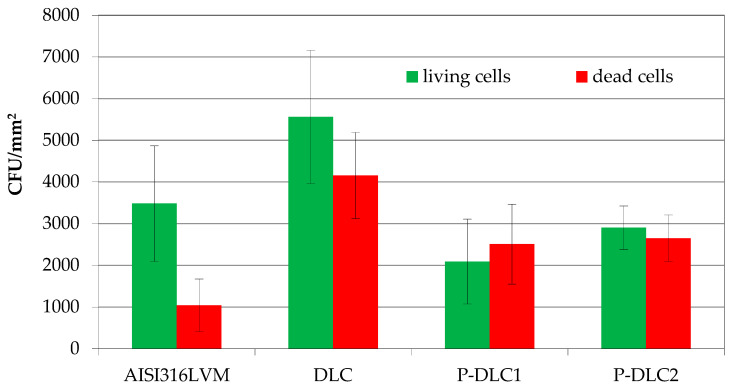
Evaluation of bactericidal/bacteriostatic effect of examined materials.

The examination of biological properties comprises three main areas of investigation: an assessment of the bacteriostatic/bactericidal effect, conducted via LIVE/DEAD staining and microscopic observations; an evaluation of the coating’s interactions with cells characteristic of the skeletal system through an osteoblast viability assay; an evaluation of interactions with cells characteristic of the vascular system through a vascular endothelium viability assay and thrombogenicity assessment. Examples of qualitative imaging data regarding the effect of doping a carbon matrix with phosphorus on model microbes are presented in Figure 9. The total number of cells present on the surface of the DLC coating is over two times higher than in the case of the bare substrate material (see Figure 10). Nevertheless, the number of dead cells comprises about 40%, while in the case of stainless steel, it is less than 25%. A slight bactericidal effect is induced by the incorporation of phosphorus into the carbon matrix. It is visible as a reduction in the number of living cells in relation to the control, as well as a high percentage of dead cells within the whole number of bacteria present on the samples’ surface. The increase in the phosphorus content does not induce significant changes in the bactericidal potential of P-DLC coatings. The bactericidal effect of P-DLC may be similar to that of black phosphorus, with the formation of reactive oxygen species [31]. The research conducted on carbon-based materials doped with phosphorus also proposes an antimicrobial effect based on electrostatic interactions. The zeta potential of *E. coli* is negative, while Chai et al. [32] reported P-doped Carbon Quantum Dots that are positively charged. In this way, cell wall disruption can be a direct effect of electrostatic bonding, which, in that paper, is also probably the case for P-DLC coatings.

**Figure 11:**
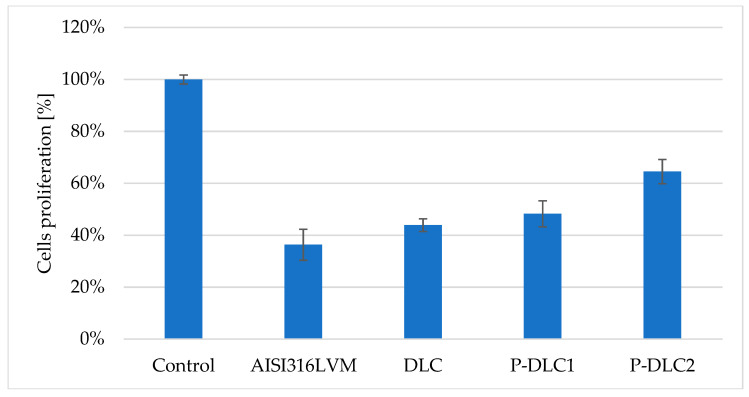

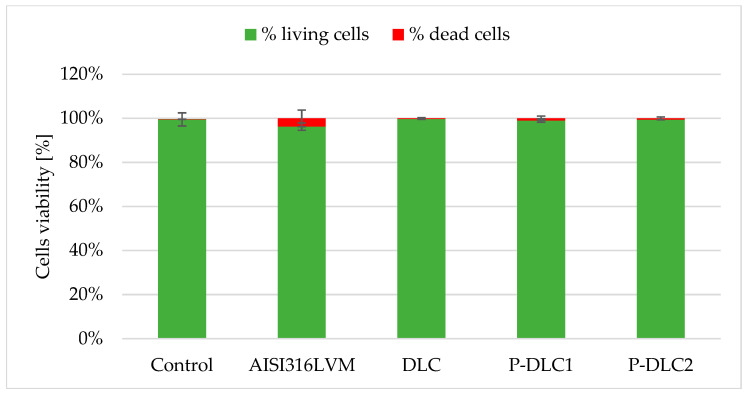
The evaluation of *Saos-2* cell proliferation on the surface of P-DLC coatings. Top—the total number of cells, expressed as a percentage of the control; bottom—the percentage of viable and dead cells.

For the sake of the initial verification of the applicability of P-DLC coatings on orthopedic implants, an assessment concerning osteoblast cells was conducted. DLC coatings improve the proliferation of *Saos-2* cells regardless of their atomic composition (Figure 11). The positive effect of admixture increases with the rising content of phosphorus. The percentage of dead cells is higher than 0.5% only in the case of a pure substrate (about 3.8%). Gao et al. [19] also obtained an increase in the proliferation of osteoblasts upon using P-DLC. In this research, only one concentration of admixture was verified, so no information on tendencies with changing amounts of the dopant element can be gleaned.

The positive effect of phosphorus on osteogenesis has already been documented, for example, in the case of black phosphorus being an admixture to hydrogel scaffolds [33,34] or in general for the sake of bone regeneration [35].

**Figure 12:**
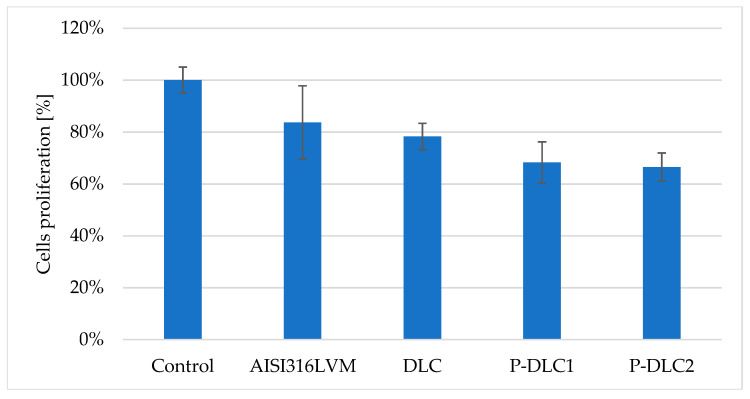

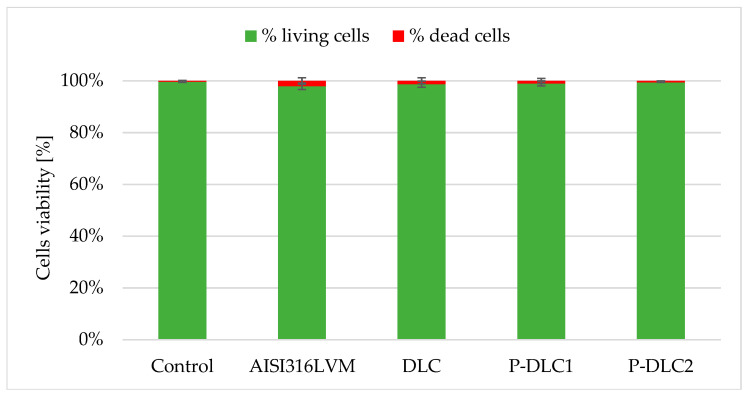
The evaluation of *EA.hy*926 cell proliferation on the surface of P-DLC coatings. Top—the total number of cells, expressed as a percentage of the control; bottom—the percentage of viable and dead cells.

The viability assessment of the vascular endothelium in contact with the examined surfaces allows us to assess important aspects of the usage of such coatings in the cardiovascular system. The *EA.hy926* cell (see Figure 12) line is less affected by the conditions of the substrate material (only about a 10% drop in proliferation in comparison to the control without any sample in comparison to over a 60% drop in the case of osteoblasts). Nevertheless, the presence of even an undoped coating results in a decrease in cell proliferation. This effect is exacerbated by the rising amount of phosphorus. For P-DLC2, the proliferation reaches only about 68% of that of the control.

A sufficient level of inorganic phosphorus is essential for osteoblast and osteocyte activity during matrix mineralization [36]. Doping with this element can regulate osteoblast proliferation and differentiation through different signaling pathways, including, e.g., the WNT, AKT, and JNK pathways [37]. In recent years, it has been shown that phosphorus has direct effects on endothelial function via dysregulation of the NO pathway. [38]. Studies have demonstrated the destructive effects of phosphate on vascular and endothelial function via disruption of the nitric oxide pathway [39]. It is believed that NO plays a very important role in physiological processes such as blood flow and pressure regulation, hormonal regulation, and neurotransmission. The examination of the viability of both *Saos-2* and *EA.hy926* cells shows the potential of P-DLC for use in orthopedic applications

**Figure 13:**
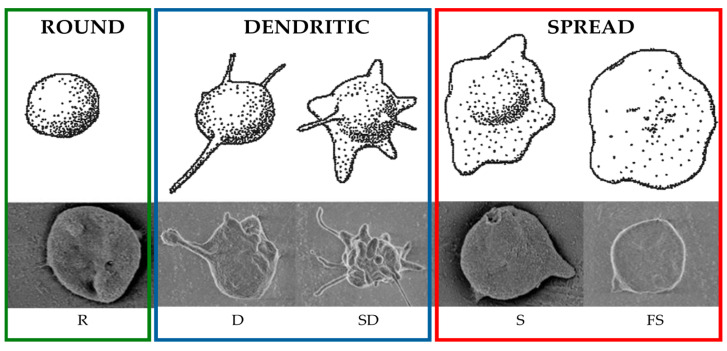
The morphological forms of activated platelets according to the Goodman scale [36]. Based on an image protected by the copyrights of John Willey and Sons (WILEY).

**Figure 14:**
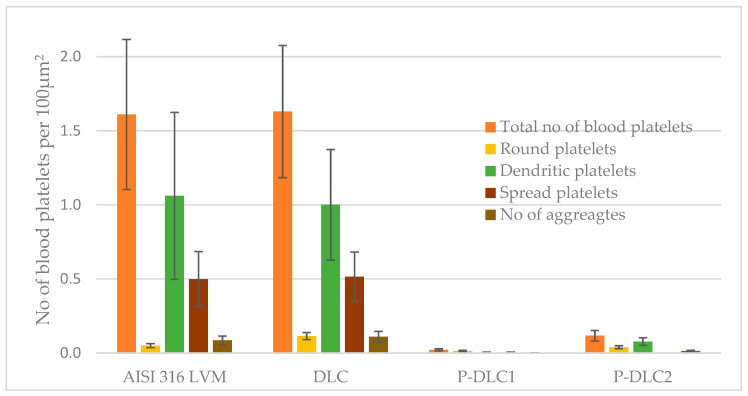
A histogram showing the number of adherent platelets on the surface of the study coatings. Round indicates unactivated platelets, Dendritic is moderately activated platelets, and Spread is highly activated platelets.

The adhesion of platelets to the test materials was assessed by counting platelets visible on SEM images of the surface of the materials after contact with blood. After contact with the artificial surface of the sample, platelets can become activated, which is visualized by a change in the shape of the platelet cytoskeleton. The degrees of platelet activation are classified by the Goodman scale [40] (see Figure 13) from single, unactivated (round) cells, to dendritic and dendritic-spread (dendritic) cells, to spread and fully spread cells with a diffuse hyaloplasm (spread). This paper categorizes platelets into unactivated (round), low-activation (dendritic), and high-activation (spread) platelets (see Figure 14). 

The platelet activation profiles of AISI316LVM and DLC coatings are almost identical. The number of moderately activated platelets is almost 2.5 times higher than the number of highly activated ones in both of these coating types, as shown in Figure 14. The total number of platelets decreases dramatically in fluoride-doped coatings, with the coating with lower P content appearing to be the most optimal in this respect. The number of adherent platelets to P-containing carbon surfaces does not seem to depend linearly on the P content. This was also shown by Kwok [41].

**Figure 15:**
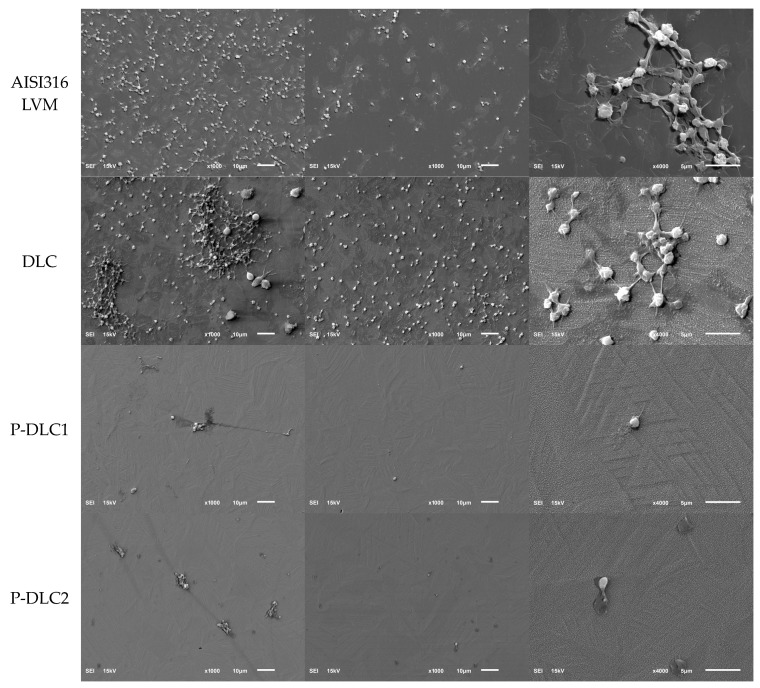
SEM images of the tested coatings after the platelet adhesion test.

Moreover, as shown in [41,42], P doping increases the polar component of the surface energy, which translates into better clotting compatibility. In the case of our coatings, the lower P content has a higher polar component of SEP and a higher ratio of polar to dispersive components. This is due to the presence of more polar groups (C=O, P=O, P-O) in P-DLC1 coatings. In the P-DLC2 coating, although it has a higher P content, the number of P-CH3 bonds increases and the polar component has a lower value. This results in increased platelet adhesion and activation. SEM images of the surface of the test samples after contact with whole blood are shown in Figure 15

## 4. Summary

P-DLC coatings of variable concentrations were successfully synthesized by means of the RF-PACVD process. Although the increase in sp2 content in the films resulted in the depletion of coating hardness, values two times higher than those of the substrate material (AISI316LVM) were achieved regardless of dopant content. The conducted biological investigation shows the high potential of P-DLC coatings for medical applications. The increasing proliferation of Saos-2 cells with higher amounts of phosphorus admixture points to the potential application of these films in cases of implants present in the skeletal system. On the other hand, significantly reduced platelet activation on P-DLC coatings makes them suitable for applications in implants in contact with blood. Also important is a small but still significant reduction in microbial colonization that would be rewarding in both cases. Among the examined biological features, the admixture of phosphorus seems troublesome only for endothelial cells. Taking all the above into consideration the potential of P-DLC is rather higher for orthopedic applications than cardiac ones.

## 5. Conclusions

Presented above is a thorough investigation of both the physicochemical and biological properties of P-DLC coatings of various dopant content, which so far has rarely been discussed in scientific publications. The novelty of this work also lies in the simultaneous and multidirectional approach toward the assessment of the potential of P-DLC films in biomedical applications on various bone or cardiovascular implants. Evaluated were the bacteriostatic/bactericidal effect, the viability of osteoblasts and vascular endothelial cell lines, and the thrombogenic character of the deposited films. The reduction in the number of bacteria on P-DLC surfaces was an unexpected result that requires further study concerning, among others, revealing the mechanism of antimicrobial action. The inhibition of microbial colonization and the improvement in the proliferation of *Saos-2* cells point to the potential future application of such coatings in orthopedics, for example, on bone plates, intramedullary nails, or hip implants. On the other hand, the thrombocompatibility of this material, despite presenting reduced proliferation of *EA.hy 926*, is still promising for applications in the blood circulatory system, like for stents. Further studies on P-DLC should cover expanding the content range of phosphorus admixture to determine the minimum amount of this dopant that would ensure a positive biological response. Another important aspect is tailoring the manufacturing process towards obtaining better adhesion to metallic medical substrates (not only AISI 316, but also alloys of titanium like Ti6Al7Nb or CoCr alloys) and the improvement of wear properties. In the future, it will be important to further examine the responses of cells in contact with P-DLC films, for example, based on research on animal models.

## Figures and Tables

**Table 1:** Element concentration of carbon, oxygen, and phosphorus in examined coatings.

	C [at%]	O [at%]	P [at%]
DLC	73.50	26.50	0.00
P-DLC 1	66.50	31.86	1.64
P-DLC 2	63.71	32.03	4.26

**Table 2:** Numerical results of deconvolution of C1 peak obtained from XPS examination of prepared samples.

	sp2 (C=C)	sp3 (C−C)	C−O	C=O	COO-R	sp3/sp2
DLC	78.10	10.37	5.90	1.80	3.87	0.13
P-DLC 1	70.80	11.50	10.23	2.47	5.00	0.16
P-DLC 2	72.05	10.25	10.00	4.55	3.10	0.14

**Table 3:** Summary of characteristic parameters withdrawn from Raman spectra of examined samples.

	D pos	G pos	G FWHM	ID	IG	ID/IG
DLC	1352.43	1594.35	68.41	236.97	246.71	0.96
P-DLC1	1360.93	1591.28	120.13	201.89	207.31	0.97
P-DLC2	1366.76	1589.83	105.20	185.31	167.93	1.10

**Table 4:** Roughness (Ra parameter) of examined coatings verified using AFM investigation.

	Scanning Area of 10 × 10 µm	Scanning Area of 1 × 1 µm
DLC	11.94 ± 1.94 nm	4.301 ± 0.56 nm
P-DLC1	21.57 ± 1.86 nm	16.99 ± 3.89 nm
P-DLC2	31.56 ± 4.16 nm	17.19 ± 2.81 nm

## Data Availability

The data presented in this study are available on request from the corresponding author due to privacy.

## References

[1] Peng Y., Peng J., Wang Z., Xiao Y., Qiu X. (2022). Diamond-like Carbon Coatings in the Biomedical Field: Properties, Applications and Future Development. Coatings.

[2] Stoica A., Manakhov A., Polčák J., Ondračka P., Buršíková V., Zajíčková R., Medalová J., Zajíčková L. (2015). Cell proliferation on modified DLC thin films prepared by plasma enhanced chemical vapor deposition. Biointerphases.

[3] Wolf D., Ley K. (2019). Immunity and Inflammation in Atherosclerosis. Circ. Res..

[4] Libby P. (2021). The changing landscape of atherosclerosis. Nature.

[5] Organisation for Economic Co-operation and Development (OECD), European Commission (2016). Hip and Knee Replacement. Health at a Glance: Europe 2019: State of Health in the EU Cycle.

[6] Jastrzebski K., Białecki J., Jastrzebska A., Kaczmarek A., Para M., Niedzielski P., Bociaga D. (2021). Induced Biological Response in Contact with Ag-and Cu-Doped Carbon Coatings for Potential Orthopedic Applications. Materials.

[7] Manninen N.K., Calderon S., Carvalho V.I., Henriques M., Cavaleiro A., Carvalho S. (2016). Antibacterial Ag/a-C nanocomposite coatings: The influence of nano-galvanic a-C and Ag couples on Ag ionization rates. Appl. Surf. Sci..

[8] Birkett M., Zia A.W., Devarajan D.K., Panayiotidis S.M.I., Joyce T.J., Tambuwala M., Serrano-Aroca A. (2023). Multi-functional bioactive silver- and copper-doped diamond-like carbon coatings for medical implants. Acta Biomater..

[9] Heaney R.P., Erdman J.W., Macdonald I.A., Zeisel S.H. (2012). Phosphorus. Present Knowledge in Nutrition.

[10] Rui S., Kubota T., Ohata Y., Yamamoto K., Fujiwara M., Takeyari S., Ozono K. (2022). Phosphate promotes osteogenic differentiation through non-canonical Wnt signaling pathway in human mesenchymal stem cells. Bone.

[11] Anselme K., Ponche A., Bigerell M. (2010). Relative influence of surface topography and surface chemistry on cell response to bone implant materials. Part 2: Biological aspects. Proc. I. Mech. E. Part H J. Eng. Med..

[12] Zareidoost A., Yousefpour M., Ghaseme B., Amanzadeh A. (2012). The relationship of surface roughness and cell response of chemical surface modification of titanium. J. Mater. Sci. Mater. Med..

[13] Kochanowska I., Chaberek S., Wojtowicz A., Marczyński B., Włodarski K., Dytko M., Ostrowski K. (2007). Expression of genes for bone morphogenetic proteins BMP-2, BMP-4 and BMP-6 in various parts of the human skeleton. BMC Musculoskelet. Disord..

[14] Regan E.M., Uney J.B., Dick A.D., Zhang Y., Nunez-Yanez J., McGeehan J.P., Claeyssens F., Kelly S. (2010). Differential patterning of neuronal, glial and neural progenitor cells on phosphorus-doped and UV irradiated diamond-like carbon. Biomaterials.

[15] Dey R., Dolai S., Hussain S., Bhar R., Pal A.K. (2018). Phosphorus doping of diamond-like carbon films by radio frequency CVD-cum-evaporation technique. Diam. Relat. Mater..

[16] Bociaga D., Kaminska M., Sobczyk-Guzenda A., Jastrzebski K., Swiatek L., Olejnik A. (2016). Surface properties and biological behaviour of Si-DLC coatings fabricated by a multi-target DC–RF magnetron sputtering method for medical application. Diam. Relat. Mater..

[17] Jelinek M., Kocourek T., Zemek J., Mikšovský J., Kubinová S., Remsa J., Kopeček J., Jurek K. (2015). Chromium-doped DLC for implants prepared by laser-magnetron deposition. Mater. Sci. Eng. C.

[18] Yetim A.F., Kovacı H., Kasapoğlu A.E., Bozkurt Y.B., Çelik A. (2021). Influences of Ti, Al and V metal doping on the structural, mechanical and tribological properties of DLC films. Diam. Relat. Mater..

[19] Gao K., Wei X., Liu G., Zhang B., Zhang J. (2020). Electrodeposition and biocompatibility of palladium and phosphorus doped amorphous hydrogenated carbon films. Chem. Phys..

[20] Batory D., Gorzedowski J., Rajchel B., Szymanski W., Kolodziejczyk L. (2014). Silver implanted diamond-like carbon coatings. Vacuum.

[21] Silverstein R.M., Webster F.X., Kiemle D.J. (2005). Spectrometric Identification of Organic Compounds.

[22] Hammad A.H., Abdelghany A.M. (2016). Optical and structural investigations of zinc phosphate glasses containing vanadium ions. J. Non-Cryst. Solids.

[23] Nayab R.S., Sasikala T., Mohan B.A., Rama M.L., Jayasankar C.K. (2017). Optical spectroscopy, 1.06μm emission properties of Nd^3+^-doped phosphate based glasses. Spectrochim. Acta Part A Mol. Biomol. Spectrosc..

[24] Corbrjdge DE C. (1956). Infra-red analysis of phosphorus compounds. J. Appl. Chem..

[25] Wan S., Hu H., Chen G., Zhang J. (2008). Synthesis and characterization of high voltage electrodeposited phosphorus doped DLC films. Electrochem. Commun..

[26] Yang C.J., Nguyen D.D., Lai J.Y. (2023). Poly(l-Histidine)-Mediated On-Demand Therapeutic Delivery of Roughened Ceria Nanocages for Treatment of Chemical Eye Injury. Adv. Sci..

[27] Lu Y., Huang G., Wang S., Xi L., Qin G., Wei J., Chen X. (2021). Pulsed laser deposition of the protective and Anti-reflective DLC film. Infrared Phys. Technol..

[28] Akaike S., Kobayashi D., Aono Y., Hiratsuka M., Hirata A., Hayakawa T., Nakamura Y. (2016). Relationship between static friction and surface wettability of orthodontic brackets coated with diamond-like carbon (DLC), fluorine- or silicone-doped DLC coatings. Diam. Relat. Mater..

[29] Domínguez-Meister S., Rojas T.C., Frías J.E., Sánchez-López J.C. (2019). Silver effect on the tribological and antibacterial properties of a-C:Ag coatings. Tribol. Int..

[30] Kwok SC H., Wang J., Chu P.K. (2005). Surface energy, wettability, and blood compatibility phosphorus doped diamond-like carbon films. Diam. Relat. Mater..

[31] Xiong Z., Zhang X., Zhang S., Lei L., Ma W., Li D., Wang W., Zhao Q., Xing B. (2018). Bacterial toxicity of exfoliated black phosphorus nanosheets. Ecotoxicol. Environ. Saf..

[32] Chai S., Zhou L., Pei S., Zhu Z., Chen B. (2021). P-Doped Carbon Quantum Dots with Antibacterial Activity. Micromachines.

[33] Miao Y., Shi X., Li Q., Hao L., Liu L., Liu X., Chen Y., Wang Y. (2019). Engineering natural matrices with black phosphorus nanosheets to generate multi-func- tional therapeutic nanocomposite hydrogels. Biomater. Sci..

[34] Raucci M.G., Fasolino I., Caporali M., Serrano-Ruiz M., Soriente A., Peruzzini M., Ambrosio L. (2019). Exfoliated Black Phosphorus Promotes in Vitro Bone Regeneration and Suppresses Osteosarcoma Progression through Cancer-Related Inflammation Inhibition. ACS Appl. Mater. Interfaces.

[35] Xu H., Liu X., Park S., Terzic A., Lu L. (2022). Size-dependent osteogenesis of black phosphorus in nanocomposite hydrogel scaffolds. J. Biomed. Mater. Res. A.

[36] Magne D., Bluteau G., Faucheux C., Palmer G., Vignes-Colombeix C., Pilet P., Rouillon T., Caverzasio J., Weiss P., Daculsi G. (2003). Phosphate is a specific signal for ATDC5 chondrocyte maturation and apoptosis-associated mineralization: Possible implication of apoptosis in the regulation of endochondral ossification. J. Bone Miner. Res..

[37] Zhou J., Wang X., Zhao L. (2019). Antibacterial, angiogenic, and osteogenic activities of Ca, P, Co, F, and Sr compound doped titania coatings with different Sr content. Sci. Rep..

[38] Stevens K.K., Denby L., Patel R.K., Mark P.B., Kettlewell S., Smith G.L., Clancy M.J., Delles C., Jardine A.G. (2017). Deleterious effects of phosphate on vascular and endothelial function via disruption to the nitric oxide pathway. Nephrol. Dial. Transplant..

[39] Szuto E., Taketani Y., Tanaka R., Harada N., Isshiki M., Sato M., Nashiki K., Amo K., Yamamoto H., Higashi Y. (2009). Dietary phosphorus acutely impairs endothelial function. J. Am. Soc. Nephrol..

[40] Goodman S.L. (1999). Sheep, pig, and human platelet-material interactions with model cardiovascular biomaterials. J. Biomed. Mater. Res..

[41] Kwok S.C.H., Wan G.J., Ho JP Y., Chu P.K., Bilek M.M.M., McKenzie D.R. (2007). Characteristics of phosphorus-doped diamond-like carbon films synthesized by plasma immersion ion implantation and deposition (PIII and D). Surf. Coat. Technol..

[42] Liu A., Zhu J., Liu M., Dai Z., Han X., Han J. (2008). Platelet adhesion on phosphorus-incorporated tetrahedral amorphous carbon films. Appl. Surf. Sci..

